# *Withania somnifera* leaf alleviates cognitive dysfunction by enhancing hippocampal plasticity in high fat diet induced obesity model

**DOI:** 10.1186/s12906-017-1652-0

**Published:** 2017-03-03

**Authors:** Shaffi Manchanda, Gurcharan Kaur

**Affiliations:** Medical Biotechnology Laboratory, Department of Biotechnology, Guru Nanak Dev University, Amritsar, 143005 Punjab India

**Keywords:** Synaptic plasticity, Cognition, Neuroprotection, Ashwagandha, Obesity

## Abstract

**Background:**

Sedentary lifestyle, psychological stress and labor saving devices in this current society often disrupts the energy gain and expenditure balance leading to obesity. High caloric diet is associated with the high prevalence of cognitive dysfunction and neuropsychiatric disorders in addition to cardiovascular and metabolic abnormalities. The present study was aimed to elucidate the potential beneficial effect of dry leaf powder of *Withania somnifera* (Ashwagandha) in preventing the cognitive decline associated with diet induced obesity.

**Methods:**

Experiments were performed on four groups of young adult female rats: [Low fat diet (LFD) rats fed on regular low fat chow, High fat diet (HFD) rats on feed containing 30% fat by weight, Low fat diet extract (LFDE) rats given regular chow and dry leaf powder of Ashwagandha 1 mg/g of body weight (ASH) and high fat diet extract (HFDE) rats fed on diet containing high fat and dry leaf powder of ASH. All the rats were kept on their respective diet regimen for 12 weeks.

**Results:**

ASH treated rats showed significant improvement in their working memory and locomotor coordination during behavioral studies as compared to HFD rats. At the molecular level, ASH treatment was observed to restore the levels of BDNF and its receptor TRKB as well as the expression of other synaptic regulators, which are highly implicated in synaptic plasticity. Further, ASH triggered the activation of PI3/AKT pathway of cell survival and plasticity by enhancing the levels of phosphorylated Akt-1 and immediate early genes viz. c-Jun and c-fos.

**Conclusions:**

ASH could be a key regulator in maintaining the synaptic plasticity in HFD induced obesity and can serve as a nootropic candidate against obesity induced cognitive impairments.

## Background

Modern lifestyle and psychological stress has drastically increased the consumption of palatable and high caloric diet, leading to increasing rates of overweight and obesity like conditions [1]. High caloric diet is associated with the risk factors for various neuropsychiatric disorders such as compulsive overeating, depression like behavior and cognitive dysfunctions in addition to metabolic and cardiac risks [2]. Recent studies have considered obesity and psychiatric comorbidities as public health problems of high relevance [3–5]. However, the cause or effect in relation to mood disorders and obesity is still a question of active interest? It is therefore important to identify the dynamics of development of such neuropsychiatric alterations and the underlying pathophysiological mechanisms in the context of obesity. Cognitive disturbances in the obese population include executive functioning such as problem solving, working memory and neuro-muscular coordination which in turn affect the social and mental ability of the obese individuals. Progress in the basic and clinical research has revealed that obesity is not only a metabolic disorder but it has been associated with low-grade inflammatory conditions leading to the immune system dysfunction. Accumulated evidence has revealed positive associations between peripheral inflammatory status and cognitive decline in obesity [6–8]. Various cross sectional and longitudinal studies have also reported the association of obesity (without any comorbid medical condition) with cognitive deficits across the lifespan from childhood to adolescence and from adulthood to old age [9, 10]. Poor performance in overweight/obese children further also suggests a bidirectional link.

In Ayurvedic medicine, *Withania somnifera* (Ashwagandha) is commonly being used for its broad spectrum of pharmacological actions. Ashwagandha is traditionally used as a rasayana (tonic) that works in a holistic manner to promote overall health and vitality. The methanolic extracts of different parts of Ashwagandha are known to exhibit therapeutic potential against various types of cardiovascular comorbidities and are also effective against hyperlipidemia and obesity [11]. Ashwagandha is known for its memory boosting and restorative functions [12–15] and is also reported to reverse loss of memory in mice model of Alzheimer’s disease by promoting the neurogenesis and growth of brain cells [16]. Similarly root extract of the plant and one of its active component withanolide A has been shown to improve spatial memory and cognitive deficits in temporal lobe epilepsy and experimental model of stroke [17, 18].

The present study was designed to investigate the potential beneficial effects of dry leaf powder of Ashwagandha in redemption of cognitive skills and neuro-muscular functions which are impaired by diet induced obesity (DIO). In Ayurvedic Traditional Medicinal System, natural products are used as dry powder or crude extract and their use is based on holistic approach. Bioactivity of a particular compound is usually not assigned. Majority of studies on Ashwagandha have evaluated its efficacy for anti-cancer and neuroprotective activities using root based ethanolic and methanolic extracts as compared to water based formulations [14, 19, 20]. Our lab is particularly using dry leaf powder and water based crude formulations of leaves as compared to root based alcoholic extracts with an aim to scientifically validate the traditional use of Ashwagandha. Moreover, the use of leaf powder and crude water extract is both eco-friendly and bio-friendly as there is no need to sacrifice the plant or use organic solvents for extraction unlike root based alcoholic formulations. Additionally, the use of powder or water based extract is convenient, safe and easy to prepare.

Rats were divided into four groups: Low fat diet (LFD) on regular chow, High fat diet (HFD) group on feed containing 30% fat by weight, Low fat diet extract (LFDE) group on regular chow and dry leaf powder of Ashwagandha (ASH) and high fat diet extract (HFDE) group on diet containing high fat and ASH. The rationale of including LFDE group in the present study was to explore any additional beneficial effect of ASH in rats consuming normal and healthy diet unlike rats fed with HFD. Interestingly, we found significant changes in some behavioral tests and their underlying molecular changes in this particular group. Therefore we presented LFDE data where we observed significant changes in this group. Post-regimen, all the rats were subjected to behavioral tests such as Novel object recognition (NOR), Rotarod and Narrow beam walk. The underlying molecular mechanism of the behavioral alterations was further explored by evaluating the expression of synaptic plasticity marker proteins such as polysialylated neural cell adhesion molecule (PSA-NCAM), neural cell adhesion molecule (NCAM) and calcium dependent synaptic regulators such as CaMKIIα and Calcineurin in hippocampus and pyriform cortex (PC) regions of the brain from these rats. The BDNF pathway of synaptic plasticity and PI3K/AKT pathway of cell survival pathway was studied in detail along with transcription factors and immediate early genes (IEGs) c-Jun and c-fos to elucidate the effect of Ashwagandha leaf powder on these effector molecules in ameliorating the impairments associated with DIO.

## Methods

### Collection and preparation of dry leaf powder of Ashwagandha

The leaves were collected from the seed raised Ashwagandha plant growing at the herbarium of the Department of Botanical and Environmental Sciences, Guru Nanak Dev University, Amritsar, India. The plant was identified and authenticated by the plant taxonomist and a voucher specimen was deposited in the herbarium for the reference purpose with deposition no. 401-24/07/1982. Our previous lab studies standardized and reported the in vivo dose of water extract of Ashwagandha (ASH-WEX), but in the present study, we used dry leaf powder of ASH. The dose of ASH powder for the current study was calculated based on our previous in vivo report [21, 22] using ASH-WEX (dry weight) at a dose of 35 mg/250 g BW of the animal. Given that 1 mg of dry weight of ASH-WEX is equivalent to 6.80 mg of dry leaf powder, so the dose corresponds to 238 mg/250 g or ~1 g/kg or 1 mg/g BW of the animal as standardized in our previous studies. The composition of ASH-WEX has been characterized and reported in our previous study [23].

### Experimental conditions

Wistar albino young female rats in the age group of 3–4 months with body weight in range of 130–150 g were used for all the experiments. All rats were caged in the group of three under controlled conditions with temperature of 25 ± 2 ˚C and dark/light cycle of 12:12 h with ad libitum access to food and water. Rats were divided into four groups: Low fat diet (LFD) on regular chow, High fat diet (HFD) group on feed containing 30% fat by weight, Low fat diet extract (LFDE) group on regular chow and dry leaf powder of Ashwagandha (ASH) and high fat diet extract (HFDE) group on diet containing high fat and ASH. The leaf powder was mixed in regular chow and high fat diet at a concentration of 1 mg/g of body weight of the animal. All the rats were kept on their respective diet regimen for 12 weeks and they were assessed for the weight gain once after every week. All procedures of animal handling and experiments were conducted in accordance with the guidelines laid down by the Institutional Animal Ethical Committee (IAEC) of Guru Nanak Dev University, Amritsar, Punjab, India and complied with arrived guidelines.

### Blood glucose and corticosterone test

The blood glucose levels of all the rats were evaluated once after every 15 day till the end of the regimen. The rats were subjected to fasting for 12–14 h before the blood glucose test. The glucose level was assessed by using glucometer by collecting small amount of blood from the animal. The corticosterone levels were analyzed from serum collected at the time of experiment post 12 weeks diet regimen, irrespective of the phase of estrous cycle using corticosterone ELISA kit by Cayman chemicals (USA) as per the manufacturer’s protocol.

### Behavioral studies

#### Rotarod test

Rotarod apparatus was an automatic motor-driven treadmill (Rotamex-5; Columbus Instruments) consisting of a 7.0 × 9.5 cm spindle diameter with a fall height of 44.5 cm from the center. The Rotarod test was performed on each animal at a fixed speed of 10 rpm for duration of 300 s. The number of falls and the time spent on the rotating rod were recorded as a measure of running performance for each trial and averaged for each group.

#### Narrow beam walk test

Narrow beam walk test was used to measure locomotor coordination and grip strength as described by Goldstein and Davis in 1990. Rats were trained on a 1 m long and 1–2 cm wide beam to transverse from a platform at one end to the dark home cage at the other end. The apparatus was placed horizontally 30 cm above the floor. The number of foot slips was recorded and the time taken to transverse the beam was recorded. The score of two successful trails was added and averaged for each animal in a group.

#### Novel Object Recognition (NOR) test

All the rats were subjected to NOR test for testing their object recognition working memory. The rats were habituated to the empty box (100 × 50 × 50 cm) for 5 min over 2 days at the start of dark phase. The activities of the rats were recorded by a video camera. Rats were allowed to explore the open box during the habituation phase for 5 min followed by the familiarization phase during which rats were placed in the box with two similar objects without having any specific odour and were allowed to explore both the objects for 5 min up to 3 days. The objects were cleaned thoroughly with 70% ethanol to ensure the absence of olfactory cues. On the test phase, the least preferred object was replaced by the novel object to check the recognition memory of the rats. Between each phase, there was a gap of 24 h. The exploration of object was defined by sniffing, licking, chewing by rats or by moving vibrissae while directing the nose towards and less than 1 cm from the object. The number of episodes and time spent in exploration of each object by each animal was recoded. The preference index between the two objects was calculated as

$$ \mathrm{Preference}\ \mathrm{Index} = \mathrm{Time}\ \mathrm{spent}\ \mathrm{in}\ \mathrm{exploring}\ \mathrm{new}\ \mathrm{object}/\mathrm{Total}\ \mathrm{time}\ \mathrm{in}\ \mathrm{exploration} $$

We further analyzed grooming and rearing behavior in rats in the open field box during NOR test to correlate the effect of stress and anxiety caused by DIO on memory and cognition. Any grooming bout of duration more than 5 s was recorded as actual grooming bout. Total time spent in grooming and rearing activity was recorded using video recording and averaged for each group.

### Immunohistochemistry

For immunostaining of PSA-NCAM in dentate gyrus (DG) region of hippocampus and pyriform cortex, experimental rats (*n* = 3) for each group were transcardially perfused with 4% paraformaldehyde (PFA) in 0.1 M phosphate buffer saline (PBS). Brains were dissected and incubated in the fixative (4% PFA) for overnight at 4 °C and subsequently cryopreserved in 20% and then 30% sucrose for 24 h each at 4 °C. Coronal sections of 35 μm were cut using cryostat microtome and treated as described in our previous study [22].

### Western blotting

Rats (*n* = 3–4 for each group) were anesthetized using thiopentone injection (1 unit per 10 g) and sacrificed to dissect out brain from the animal. Pyriform cortex (PC) and hippocampus regions were dissected out and lysed in lysing buffer using tissue homogenizer and centrifuged at 5000 rpm for 10 min at 4 ˚C. Supernatant was collected after centrifugation and used for protein estimation by Bradford method. 50 μg of protein from each sample was mixed with 6X sample buffer. Protein samples were resolved in 7–10% SDS-PAGE as per their molecular weight for better resolution, followed by transfer onto a 0.45 μm pore size PVDF membrane (Hybond-P from Amersham Biosciences UK Limited) using the semi-dry Novablot system (Amersham Biosciences UK Limited) at 90 mA for 90 min. Transferred membranes were then blocked with 5% skimmed milk in 0.1% TBS-Tween 20 for 2 h. Further, membranes were probed with mouse monoclonal anti-PSA-NCAM (1:1500), anti-NCAM (1:2000), anti CaMKIIα (1:2000), anti CaN (1:2000) anti-phospho Akt-1 (1:2000), (Sigma-Aldrich St. Louis, MO, USA), anti-c-Jun (1:2000), anti-c-fos (1:2000) (Santa Cruz Biotechnology Inc., USA), for overnight at 4 °C. This was followed by three washing with 0.1% TBS-Triton-X 100 of 10 min each and incubation with HRP-labeled anti-mouse IgM (1:2000) or anti-rabbit IgG (1:5000) or anti-mouse IgG (1:5000) secondary antibodies (Genei) for 2 h at 25 ˚C. The remaining procedure was followed as described in our previous study [22].

### mRNA expression analysis by Quantitative Real time PCR (RT-PCR)

Total RNA was extracted from the cells by TRI reagent (Sigma-Aldrich) according to manufacturer’s instructions. For cDNA synthesis, 5 μg of RNA, 1 μl of 250 ng (N6) random hexamer (Applied Biosystem), 1 μl of dNTP mix (10 Mm of each dNTP (Amersham), nuclease free sterile water upto 12 μl, 4 μl 5X first strand buffer, 2 μl of 1 M DTT, 1 μl of of recombinant ribonulease inhibitor (40 units/ μl) (Invitrogen), 1 μl of 200 U of M-MLV reverse transcriptase (Invitrogen) were added for 20 ul reaction volume. 50 μg of cDNA was then used to amplify a gene of interest using StepOne Plus Real Time PCR system (Applied Biosystems). Each 5 μl reaction mixture comprised of 2.5 μl of 2X SYBR green Master Mix, 1 μl of 20× predesigned Primer mix (Applied Biosystem) and of 1 μl water and 1 μl (50 μg) cDNA. GAPDH was used as an endogenous control for each gene of interest. The relative gene expression of each gene was calculated by ‘Livak method’ and represented as 2 − ΔΔCt and final gene expression as 2 − ΔΔCt ± SEM.

### Statistical analysis

Values were expressed as mean ± SEM of the values obtained from at least three independent experiments. The Sigma Stat for Windows (version 3.5) was used to analyze the results by Student’s *t*-test, one -way ANOVA, two-way ANOVA (Holm-Sidak post hoc method), in order to determine the significance of the mean values.

## Results

### Body weight, blood glucose and corticosterone

**Fig. 1:**
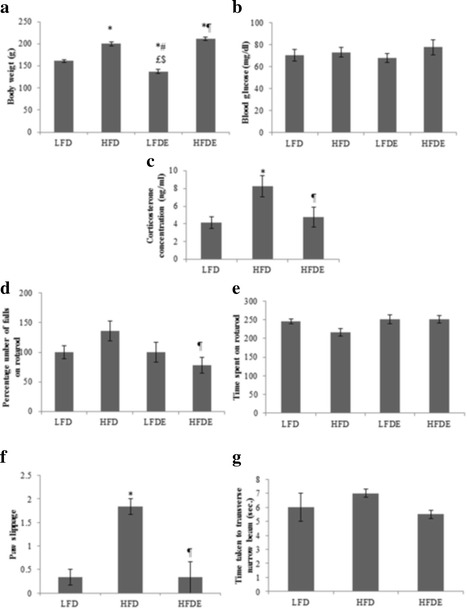
Comparison of body weight, blood glucose, corticosterone and analysis of locomotor coordination tests. HFD regimen significantly increase the body weight in HFD and HFDE rats (**a**) compared to control rats. A significant reduction in the body weight was found in LFDE rats (**a**). Blood glucose levels remained unchanged between the groups (**b**). Corticosterone levels were significantly increased in HFD rats and restored to control levels in HFDE rats (**c**). During rotarod test, ASH treated HFD rats performed better than HFD rats during as assessed by latency to fall (**d**) and time spent (**e**) on rotarod. HFDE rats also performed better than HFD rats during narrow beam walk test as assessed by the paw slippage (**f**) and time taken (**g**) to transverse the beam indicating their intact locomotor functions. Values are expressed as ± SEM, *p < 0.05 LFD vs. HFD, LFDE and HFDE, ^#^ *p* < 0.05 LFDE and HFD rats, ^¶^ *p* < 0.05 HFDE vs. HFD rats, ^$^signifies highly significant levels. Holm sidak post-hoc test after one-way ANOVA

A gradual increase in the body weight was observed in rats fed with their respective diets and on completion of diet regimen, HFD rats weighed 24% more than the LFD rats. Interestingly, the percentage weight in LFDE rats was reduced to 15% compared to LFD alone group, whereas, body weight was similar in HFD and HFDE groups (Fig. 1a). Further, the blood glucose levels showed no significant change between the groups (Fig. 1b). Corticosterone levels were significantly higher in HFD rats compared to LFD rats and supplementation of ASH in HFDE group normalized the up regulated levels of corticosterone to basal levels (Fig. 1c).

### Behavioral studies

#### Locomotor coordination

Animals were tested for their locomotor skills on rotarod and were accessed for their latency to fall from the cylindrical rod and the time spent on the rod. HFD rats showed maximum number of falls and spent significantly less time (*p* ≤ 0.05) on rotating rod compared to other three groups indicating impaired locomotor function in these rats (Fig. 1d and e). However, LFDE and HFDE rats showed lower latency to fall from the rod (*p* ≤ 0.05), indicating reversal to normal locomotor function (Fig. 1d and e). During narrow beam walk test, rats were assessed for the time taken to transverse the narrow beam and the frequency of paw slippage. HFD rats showed maximum paw slippage (*p* ≤ 0.01) and took longer time to transverse the beam compared to LFD rats (Fig. 1e and f). HFDE rats transversed the beam very fast with negligible paw slippage with score of less than 1 (*p* ≤ 0.01) (Fig. 1f and g), thus indicating their strong grip over the beam and intact locomotor function.

#### Novel object recognition test

**Fig. 2:**
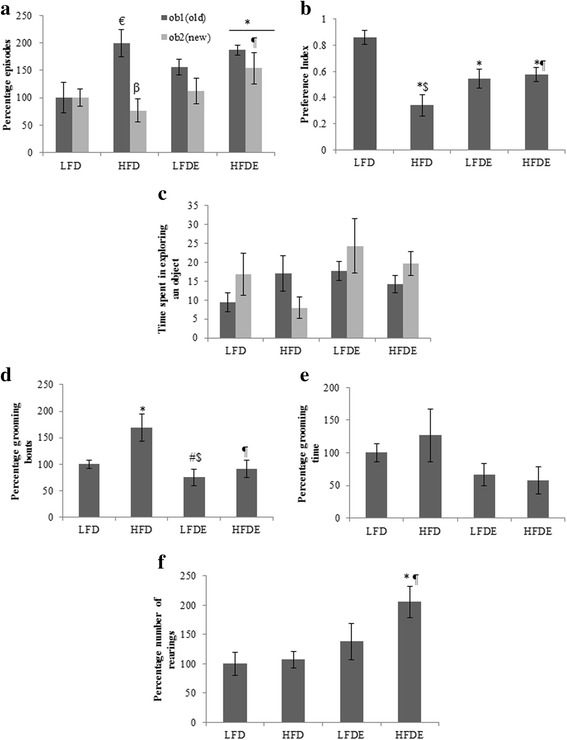
Analysis of various parameters during recognition memory test. Novel object recognition test performance is shown in a-f. HFD rats showed significantly less number of episodes in exploring the new object than old one (**a**) compared to control rats, whereas HFDE rats spent more time and episodes in exploring the new object as compared to HFD rats (**a** and **c**) indicating their intact recognition memory. Significant difference in the preference index among HFD and HFDE group rats indicates the preference of the ASH treated HFD rats for the novel object (**b**). HFD rats spent more time in grooming with significantly higher percentage of grooming bouts compared to control rats (**d** and **e**) indicating their high stress levels. No significant change was found in the number of rearings among all the groups except HFDE rats with highest rearing activity (**f**). Values are expressed as ± SEM, *p < 0.05 LFD vs. HFD, LFDE and HFDE, ^#^ *p* < 0.05 LFDE and HFD rats, ^¶^ *p* < 0.05 HFDE vs. HFD rats, ^β^ *p* < 0.05 old vs new within a group, ^€^ *p* < 0.05 LFD vs HFD within old, ^$^signifies highly significant levels. Holm sidak post-hoc test after one way and two-way ANOVA

Rats were subjected to NOR task to test their recognition memory and learning. HFD rats showed impaired memory in recognizing the novel object compared to LFD, LFDE and HFDE counterparts as evident from their significantly higher number of episodes and time spent in exploring the old object compared to the novel object (*p* ≤ 0.05) indicating their impaired recognition memory (Fig. 2a and c). On the contrary, HFDE rats spent more time (*p* ≤ 0.05) in exploring the novel object over the old one which was better than LFD group, thus indicating their intact memory (Fig. 2c). Likewise, these rats showed higher preference for the novel object (*p* ≤ 0.01) with preference index (PI) score of more than 0.5, further supporting their intact recognition memory whereas HFD rats failed to show their preference for the novel object and had PI score of less than 0.5 (Fig. 2b).

Interestingly, HFD rats showed highest frequency of grooming bouts (*p* ≤ 0.01) and spent maximum time in grooming during NOR test indicating high stress/anxiety levels of these rats compared to the other groups (Fig. 2d and e). Compared to HFD rats, HFDE rats showed normal grooming behavior with very low frequency which was even better than LFD and LFDE groups indicating their low stress levels (Fig. 2d and e). Additionally, rats were assessed for their rearing behavior also, wherein HFD rats showed comparable frequency of rearing to LFD rats, whereas, LFDE and HFDE rats showed higher frequency of rearing (*p* ≤ 0.05) indicating their interest in exploring the empty box (Fig. 2f).

#### Synaptic plasticity and cell survival markers

**Fig. 3:**
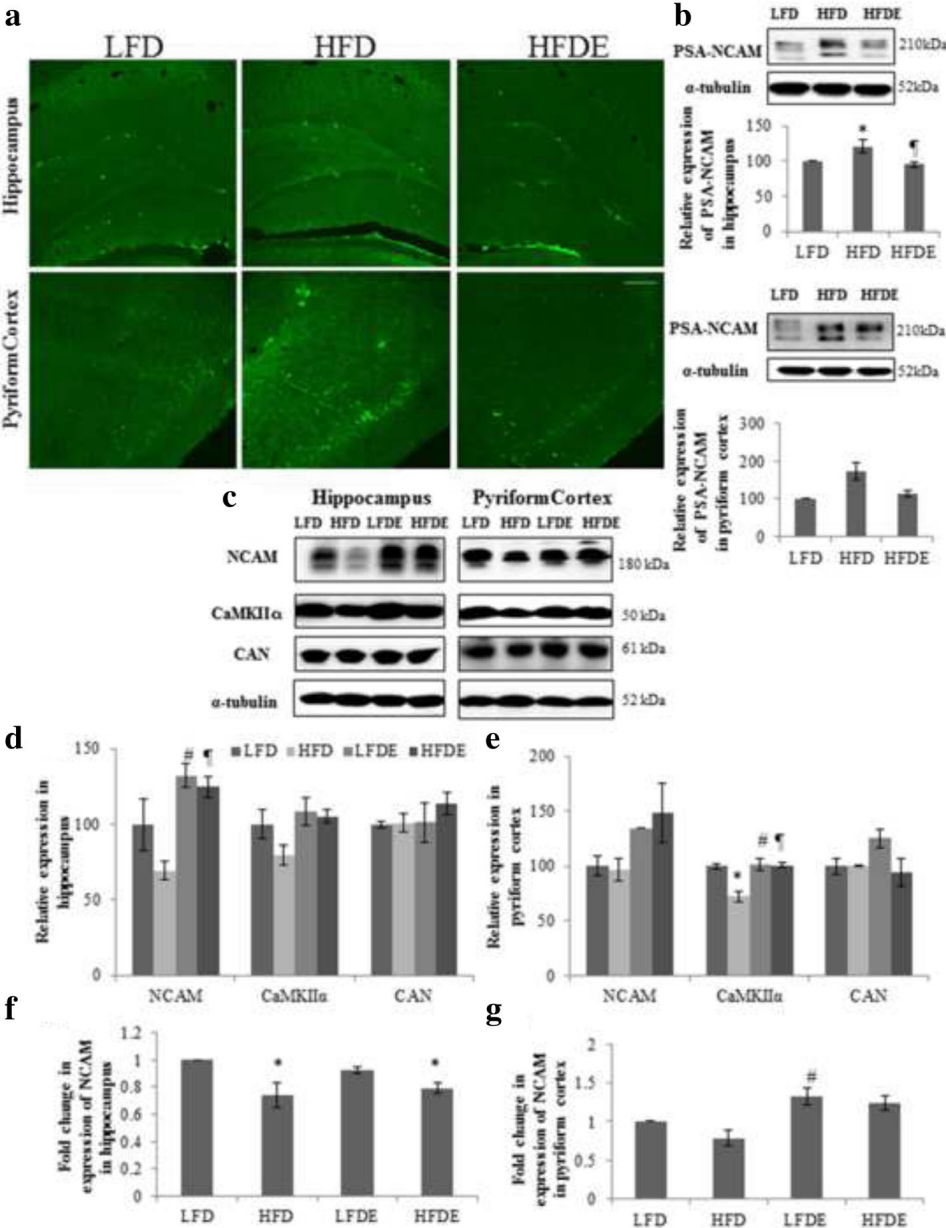
Quantitative analysis of synaptic plasticity markers. Representative immunohistochemical images of PSA-NCAM (**a**), representative western blot images and densitometric analysis of PSA-NCAM (**b**), representative protein bands and densitometric analysis of NCAM, CaMKIIα, and CAN (**c**, **d** and **e**) and histograms representing fold change in expression of NCAM (**f** and **g**) in hippocampus and pyriform cortex (PC) regions of rat brain among the three groups (*n* = 3 for each group). *p < 0.05 LFD vs. HFD, LFDE and HFDE rats, ^#^ *p* < 0.05 LFDE vs. HFD rats, ^¶^ *p* < 0.05 HFDE vs. HFD rats. Holm-sidak test after one-way ANOVA. Scale bar-200 μm

Hippocampal synaptic plasticity and spatial memory are among very well reported brain functions which are known to get seriously impaired by genetic and DIO. The expression of some plasticity markers involved in synaptic transmission and plasticity were studied from hippocampus and PC regions. HFD rats showed increase in the expression of PSA-NCAM compared to the LFD rats and it was seen to be normalized with ASH treatment in HFDE rats (*p* ≤ 0.05) in both the brain regions (Fig. 3a and b). The expression of NCAM was down regulated in HFD rats, whereas it was significantly up regulated in LFDE and HFDE rats in both the brain regions at both the transcriptional and translational levels (Fig. 3c, d–g). Further, the expression of calcium and calmodulin dependent protein kinase, CaMKIIα was reduced significantly in HFD rats in both the brain regions which was normalized in LFDE and HFDE rats by ASH (Fig. 3c second panel, d and e). The expression of protein phosphatase CAN remained unaffected in hippocampus region while it was down-regulated in PC region in HFD rats (Fig. 3c third panel, d and e). ASH treatment was seen to prevent the decrease in expression of CAN in PC region (Fig. 3c third right panel and e).

**Fig. 4:**
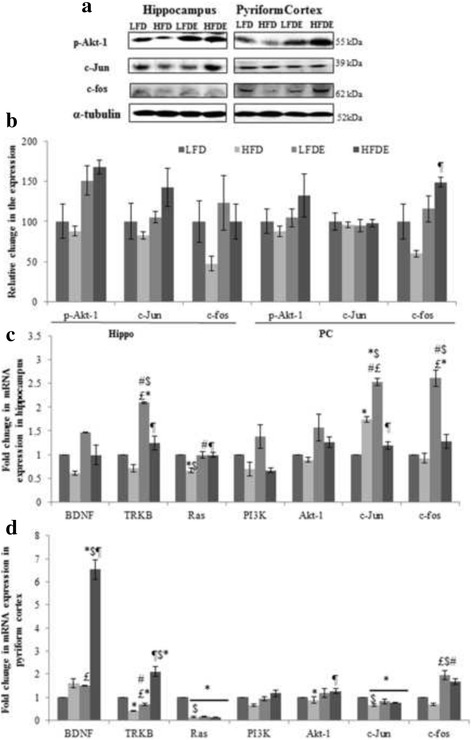
Quantitative mRNA expression analysis of genes involved in BDNF mediated synaptic plasticity and cell survival. Representative immunoblots (**a**) and densitometric analysis (**b**) of markers involved in cell survival such as Akt-1, c-Jun and c-fos in hippocampus and pyriform cortex regions of rat brain. Quantitative mRNA expression of BDNF, TRKB, RAS, PI3K, Akt-1, c-Jun and c-fos in hippocampus (**c**) and pyriform cortex (**d**) regions of rat brain among the four groups (*n* = 3 for each group).*p < 0.05 LFD vs. HFD, LFDE and HFDE rats, ^#^ *p* < 0.05 LFDE vs. HFD rats, ^£^ *p* < 0.05 LFDE vs. HFDE rats, ^¶^ *p* < 0.05 HFDE vs. HFD rats, ^$^signifies highly significant levels. Holm-sidak test after one-way ANOVA

Phosphorylation of Akt at ser 473 is known to be important event in PI3K mediated synaptic plasticity and memory consolidation [24]. Phosphorylation of Akt-1 was reduced in HFD rats while it was recovered in LFDE and HFDE rats in both the brain regions (Fig. 4a and b). The expression of IEGs such as c-jun and c-fos was down-regulated in HFD rats, while it was up regulated in LFDE and HFDE rats in hippocampus region (Fig. 4a left panel and b). In PC region, the expression of c-Jun showed no change between the groups, whereas, c-fos expression was markedly decreased in HFD rats and was up regulated in LFDE and HFDE rats (*p* ≤ 0.05) (Fig. 4a right panel and b).

#### Quantitative Real-time PCR expression of brain derived neurotrophic factor and PI3/AKT pathway

The expression of BDNF and TRKB was found to be down regulated in HFD rats while ASH treatment was observed to compensate for the decreased expression in LFDE and HFDE rats in hippocampus region (Fig. 4c). The phosphotyrosine residues of TRKB receptor functions as binding site for recruiting certain signaling and scaffolding proteins that in turn activate Ras (a family of guanosine nucleotide binding proteins that control intracellular signaling events). In HFD rats, the mRNA expression of Ras was significantly down regulated in hippocampus region, which on treatment with ASH was normalized to basal levels in LFDE and HFDE rats (*p* ≤ 0.01) (Fig. 4c). Ras further activates PI3K/AKT pathway in addition to other signaling networks. The expression of PI3K was down regulated in both HFD and HFDE rats, whereas, in LFDE rats, it was found to be markedly increased in hippocampus region (Fig. 4c). Real time mRNA expression of total Akt-1 was down regulated in HFD rats, while it was up regulated in LFDE and HFDE rats in hippocampus region (Fig. 4c). The mRNA expression of c-Jun was increased 1.5 fold in HFD rats and was further increased to 2.5 fold in LFDE rats compared to LFD group, whereas, in HFDE rats, the expression was normalized to basal levels in hippocampus region (*p* ≤ 0.01) (Fig. 4c). The expression of c-fos was slightly down regulated in HFD rats, whereas 1.5–2.5 fold increase in the expression of this IEG was observed in LFDE and HFDE rats from hippocampus and PC regions (Fig. 4c and d).

In PC region, a slight increase in expression of BDNF was observed in HFD and LFDE rats, whereas in HFDE rats, a 6-fold increase was found in its expression (Fig. 4d). The expression of TRKB was down regulated in HFD rats, while it was significantly increased in LFDE and HFDE rats (Fig. 4d). A marked decrease in the expression of Ras was observed in HFD, LFDE and HFDE groups compared to LFD rats (*p* ≤ 0.001) (Fig. 4d). The expression of PI3K was significantly down regulated in HFD rats and was normalized in LFDE and HFDE rats (Fig. 4d). No change in the expression of Akt-1 was found among HFD, LFDE and HFDE rats compared to LFD group (Fig. 4d) in this brain region. The expression of c-Jun was decreased in HFD, LFDE and HFDE groups compared to LFD group (Fig. 4d).

## Discussion

The present study provides evidence that 12 weeks of HFD regimen initiated in young adult female rats caused no hyperglycemic condition but showed significant impairment in their memory and locomotor functions. These observations are supported by previous studies reporting that HFD feeding developed no hyperglycemia in females as compared to their age matched male counterparts [25]. Estradiol levels in female mice have been proposed to be one of the reasons for the absence of hyperglycemia as compared to male mice [26]. However, in rats, no change in the estradiol levels has been reported in obese group of both the sexes. Based on previous studies data, it may be suggested that hyperglycemic condition in males is attributed to their less energy expenditure as compared to females on HFD despite the similar energy intake requirements in both males and females [25, 27]. Interestingly, ASH consumption was observed to manage body weight gain in rats on regular chow diet, whereas, HFD regimen masked the weight reducing effect of ASH in HFDE rats. A recent clinical study on root extract of Ashwagandha has reported the body weight management in adults under chronic stress by reducing the serum cortisol, physiological and psychological stress thereby improving the eating behavior [28].

### ASH prevented the memory related cognitive impairments and maintained locomotor coordination in HFD rats

During locomotor coordination studies, HFD rats showed both lack of interest and strength in performing these tasks as evident from higher number of falls and less time spent on the rotating rod. ASH treated LFDE and HFDE rats (Fig. 1d and e) performed as good as LFD rats, thus indicating their normal neuromuscular coordination. During narrow beam walk test, HFD rats were observed to have poor balance over the narrow beam and showed high percentage of paw slippage compared to LFD and HFDE rats (Fig. 1f). Further HFD rats took longer time to transverse the entire beam, whereas, LFDE and HFDE rats spent time almost equal to LFD group to transverse the beam (Fig. 1g) indicating their intact locomotor functions. These observations from the behavioral studies indicated that HFD regimen caused locomotor and neuro-muscular dysfunction and ASH have the potential to improve motor performance and body balance in rats on HFD regimen.

During novel object recognition task, HFD rats showed impairment in their recognition and working memory as is suggested by higher number of episodes in exploring the old object over the novel one (Fig. 2a) and less time spent in exploring the novel object (Fig. 2c). Both LFDE and HFDE rats performed better as they spent more time in exploring the novel object compared to the old one (Fig. 2c). These observations were strongly supported by the PI score of the rats (Fig. 2b). The HFD rats showed least preference for the novel object (PI ≤ 0.5), whereas ASH supplemented rats showed more preference for the novel object (PI ≥ 0.5) (Fig. 1b). A PI score of less than 0.5 shows no preference for any of the objects and more than 0.5 score shows preference for the novel object [29]. Further, we studied grooming behavior in these rats during NOR test and observed that HFD rats showed higher percentage of grooming bouts, whereas LFDE and HFDE groups showed similar percentage of grooming bouts to LFD animals (Fig. 2d). Grooming is a nonspecific behavior that serves a broad range of purposes [30]. In rodents, grooming is an important behavioral repertoire and is associated with their adaptation to stress conditions as well as to self-cleaning [31]. Rodents generally follow a well ordered and sequential grooming pattern starting from rostrum up to tail under low stress conditions. Conversely, animals with high stress/anxiety levels display fragmented grooming pattern [31, 32]. In other words, self-grooming with non-structured pattern is considered as a marker of increased anxiety like behavior. In our study, HFD rats spent maximum time in grooming with interrupted and fragmented pattern paralleled by high number of grooming bouts, thus indicating their anxiogenic behavior. However, ASH supplemented rats showed least grooming activity coupled to a sequential grooming pattern as compared to HFD rats (Fig. 2e), thus suggesting the anxiolytic potential of ASH powder.

These observations are also supported by the rearing activity of the ASH supplemented rats as these were observed to show maximum rearing episodes compared to LFD and HFD rats (Fig. 2f). Rearing is the innate exploratory behavior found in rodents under most of the environmental conditions and is considered as a marker of environmental novelty that can be used to measure hippocampal learning and memory task [33]. Increased levels of corticosterone also support the higher stress levels and metabolic abnormalities in rats fed with HFD regimen, whereas, ASH feeding restored the basal levels of the stress hormone and the metabolic state in HFD rats (Fig. 1c). Since serum corticosterone levels were analyzed irrespective of the phase of estrous cycle, it may be possible that the different phases of estrous cycle have also contributed to these changes. However, a recent study carried out on high fat diet induced obesity has reported an increase in corticosterone levels in young adult male and female rats [34]. Several other studies in HFD induced obesity model have reported increased levels of corticosterone and their metabolites in obese rats which along with insulin increased the fat stores and contributed to obesity due to high caloric intake [35, 36]. Both basic and clinical research has reported memory retention and memory enhancing role of aqueous and alcoholic formulations from root of Ashwagandha [37–39]. Aqueous leaf extract of Ashwagandha has been recently reported by our lab to improve memory retention functions by reducing the cellular stress via ameliorating the synaptic plasticity and cell survival pathways in acute sleep deprived rats [23]. Based on these observations, it may be suggested that ASH supplementation reduced the stress levels and exhibit nootropic effect in HFD induced obese rats.

### ASH suppressed the change in synaptic plasticity markers induced by high fat diet regimen

Cognitive impairments are known to be the outcomes of alterations in hippocampal synaptic plasticity thus affecting learning performance and memory functions. Experiments to discern the underlying molecular mechanism of these alterations revealed up regulation in the expression of PSA-NCAM in hippocampus and PC regions of brain with HFD regimen (Fig. 3a and b). It has been reported that chronic restraint stress up-regulates the expression of PSA in DG and pyriform cortex regions of brain while inhibiting the process of neurogenesis [40, 41]. The enhanced polysialylation in HFD animals may be a neuroprotective mechanism in stress vulnerable neuronal circuits promoting the structural recovery and integrity. However, ASH treatment compensated for the stress induced increase in the expression of PSA-NCAM in HFDE rats (Fig. 3a and b). It has been reported that PSA is required for the basal synaptic transmission of new neurons but not in synaptic potentiation, which is solely dependent on NCAM expression [42]. A very recent study has reported that increased expression of NCAM1 with donepezil (acetylcholinesterase inhibitor) and aniracetam (nootropic) improved spatial memory in status epilepticus rats [43]. The poor recognition memory of HFD fed rats might be attributed to decreased expression of NCAM and ASH treatment significantly increased the mRNA and protein expression of NCAM (Fig. 3c, d–g), thereby improving the performance of LFDE and HFDE rats in memory task observed in the current study.

Further, the expression of CaMKIIα was evaluated as it is highly implicated as an initial signaling event in long term memory storage and its constitutive exposure to neurons is known to produce synaptic enhancement. It has been reported that there is reduction in the level of total CaMKIIα in DG and CA1 regions of hippocampus in obese zucker indicating the impairment of synaptic potentiation in hippocampus specifically in the CA1 region [44]. In the present study, the expression of CaMKIIα was significantly down regulated in rats on HFD regimen in both the brain regions (Fig. 3c, d–e) indicating the impairment in calcium mediated synaptic plasticity. ASH supplementation in LFDE and HFDE rats up regulated the expression of CaMKIIα in both the brain regions (Fig. 3c, d–e) indicating the conditions, permissive for synaptic enhancement. Another important regulator of synaptic plasticity is calcineurin and it has been reported that overexpression of calcineurin activate long term depression [45, 46]. In our study, we found no change in the protein expression of CAN between all the treatment groups compared to LFD rats in hippocampus region (Fig. 3c and d), whereas, in PC region, it was decreased in HFD rats and remained unchanged in LFDE and HFDE (Fig. 3c and e). This reduction in PC region could be due to a compensatory mechanism in HFD rats against the reduced expression of total CaMKIIα and due to divergent nature of neurons existing in the two regions.

Based on the changes in the synaptic plasticity molecules in the current study, it may be suggested that poor performance of HFD rats in NOR and locomotor coordination tests could be due to their impaired synaptic function. However, ASH supplementation restored the synaptic function by maintaining the optimal expression of NCAM and its polysialylated form, CaMKII (all implicated in synaptic transmission and learning and memory) thereby improving the performance of rats during the behavioral tasks.

### BDNF/PI3K/AKT pathway mediated modulation of synaptic plasticity in high fat diet regimen

High caloric diet is known to impair learning and memory by modulating synaptic plasticity and neurogenesis accompanied by reduction in the BDNF levels and dendritic spine density in hippocampus of the rats. Long term HFD feeding reduced the BDNF and its tyrosine receptor TRKB levels in hippocampus region of the brain (Fig. 4c) indicating that reduction in the synaptic plasticity in HFD rats may be mediated by the BDNF pathway. Several studies have reported that impaired cognitive functions and hippocampal neurogenesis in middle aged rats and in young offspring’s of the mother under high caloric regimen are mediated by reduced BDNF levels [47, 48]. Interestingly, ASH supplementation recovered the BDNF and TRKB levels in the LFDE and HFDE rats (Fig. 4c), indicating the restoration of the hippocampal function of learning and memory and synaptic plasticity. Treadmill exercise has been recently reported to improve the cognitive functions in high fat diet induced obese mice by improving the levels of BDNF and TRKB in hippocampus region of brain [4]. The better performance of ASH supplemented rats in learning and memory tasks may be due to their enhanced BDNF and TRKB levels in the hippocampus. In PC region, the expression of BDNF was increased in all the groups compared to LFD rats (Fig. 4d) which could be due to differential expression and synthesis of this neurotrophic factor in the two brain regions as supported by previous studies [49, 50]. The expression of TRKB was decreased in HFD rats and ASH supplementation restored the expression of TRKB to normal levels (Fig. 4d) similar to hippocampus region.

**Fig. 5:**
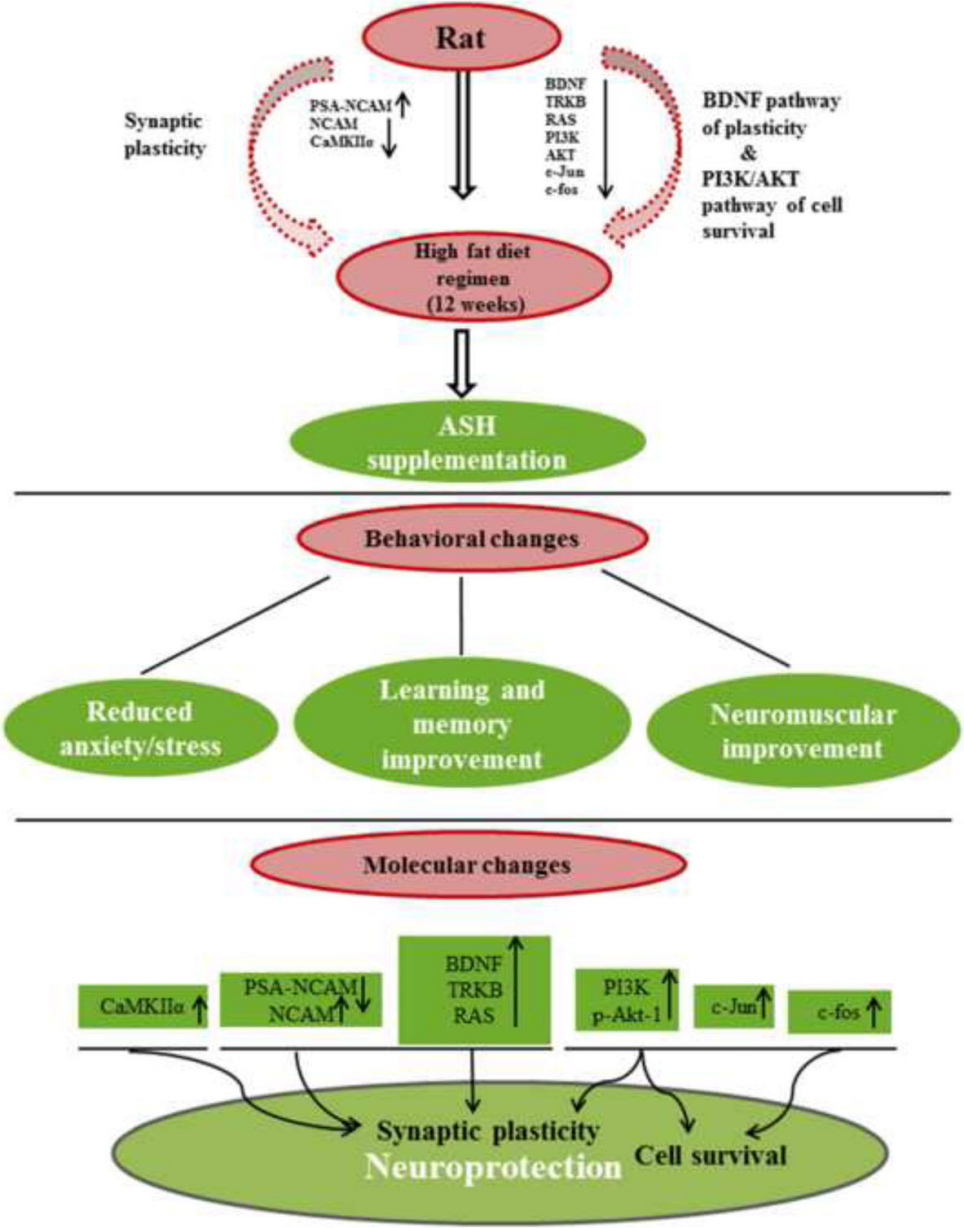
Schematic representation of the study. HFD regimen ameliorated key synaptic and cell survival pathways and ASH supplementation reduced stress levels and may have prevented memory and neuro-motor impairments caused due to HFD regimen. ASH exhibits a pleotropic neuroprotective phenomenon by targeting key pathways such as modulating BDNF mediated synaptic plasticity and cell survival in discrete brain regions, thereby promoting overall health and vitality

Neurotrophins are known to promote neuronal survival by Ras dependent activation of PI3 Kinase which in turn phosphorylates Akt to promote cell survival and inhibit apoptosis. PI3K/AKT is the principal signaling pathway that allows the cell to withstand apoptotic stimulus and promote cell survival. Down-regulation in the expression of PI3K gene in HFD rats (Fig. 4c and d) may be due to the inhibition of cell survival signal, which was restored to LFD levels in ASH supplemented rats (Fig. 4c) thus, suggesting the activation of cell survival signaling events in these rats. Likewise, the gene expression level of total and phosphorylated Akt-1 was down regulated in HFD rats further confirming the suppression of cell survival signal in these animals (Fig. 4a–d). ASH supplementation enhanced the expression of total and phosphorylated Akt-1 in LFDE and HFDE rats in both the brain regions, thereby promoting the survival conditions in the neuronal cells. Similarly, the expression of c-Jun and c-fos (IEGs in the cell survival mechanism) was suppressed in HFD rats (Fig. 4a–d), while ASH supplementation restored the expression levels of these transcription factors, specifically in hippocampus region (Fig. 4a left panel and b). In PC region, we observed activation of cell survival molecules but the changes were more prominent in hippocampus region. The possible reason for this differential response might be due to profound modulation of structural and functional plasticity and subsequent activation of survival molecules in hippocampus region as compared to the PC region. Stress induced activation of glucocorticoids are known to strongly inhibit the hippocampal BDNF expression in HFD rats [51, 52]. So it may be suggested that reduction in the BDNF expression may be linked to enhanced expression of corticosterone in HFD rats. BDNF enhances efficiency of synaptic transmission and hippocampal synaptic plasticity by activating CaMKII in hippocampus region thereby improving the cognitive performance [53]. In conclusion, it may be suggested that ASH treatment improved the memory and learning based functions by promoting the activity dependent hippocampal plasticity and cell survival and by reducing the cellular stress (schematic representation of the possible molecular pathways in ASH mediated activity in HFD rats in Fig. 5).

## Conclusions

Obesity has been associated with the progressive cognitive decline and impairments in the synaptic plasticity and potentiation by interplay between neurotropic factors and oxidative stress [54]. Ashwagandha as an ayurvedic formulation have been known for decades for its rejuvenating and life prolonging effects thereby promoting health and survival. The current data may suggest that HFD regimen impaired recognition memory and locomotor balance and ASH supplementation improved the behavioral outcomes of learning and memory as well as locomotor coordination in Wistar rats, thereby promoting neuroplasticity. The possible mechanism of ASH may be via BDNF mediated synaptic modulation and AKT mediated cell survival (Fig. 5). These observations validate the traditional use of Ashwagandha as nootropic candidate which seems to play a key role in maintaining the synaptic plasticity in HFD induced obesity and may serve as a potential candidate against obesity induced cognitive impairments.

## Abbreviations

ASH: Dry leaf powder of Ashwagandha
DIO: Diet induced obesity
HFD: High fat diet
HFDE: High fat diet extract
LFD: Low fat diet
LFDE: Low fat diet extract
NOR: Novel object recognition
SEM: Standard error of mean
